# Fe–Ni/MWCNTs Nano-Composites for Hexavalent Chromium Reduction in Aqueous Environment

**DOI:** 10.3390/molecules28114412

**Published:** 2023-05-29

**Authors:** Zeyu Kang, Hui Gao, Xiaolong Ma, Xiaodong Jia, Dongsheng Wen

**Affiliations:** 1 School of Chemical and Process Engineering, University of Leeds, Leeds LS2 9JT, UK; 2 School of Aeronautic Science and Engineering, Beihang University, Beijing 100191, China; 3 School of Engineering and Design, Technische Universität München, 85747 Garching, Germany

**Keywords:** environment remediation, hexavalent chromium, catalytic reduction, multi-walled carbon nanotube, kinetics modelling

## Abstract

A novel Cr (VI) removal material was designed and produced comprising multi-walled carbon nanotubes (MWCNTs) as a support with a high specific surface area and the loaded Fe–Ni bimetallic particles as catalytic reducing agents. Such a design permits the composite particle to perform the adsorption, reduction, and immobilisation of Cr (VI) quickly and efficiently. Due to MWCNTs’ physical adsorption, Cr (VI) in solution aggregates in the vicinity of the composite, and Fe rapidly reduces Cr (VI) to Cr (III) catalysed by Ni. The results demonstrated that the Fe–Ni/MWCNTs exhibits an adsorption capacity of 207 mg/g at pH = 6.4 for Cr (VI) and 256 mg/g at pH 4.8, which is about twice those reported for other materials under similar conditions. The formed Cr (III) is solidified to the surface by MWCNTs and remains stable for several months without secondary contamination. The reusability of the composites was proven by retaining at least 90% of the adsorption capacity for five instances of reutilization. Considering the facile synthesis process, low cost of raw material, and reusability of the formed Fe–Ni/MWCNTs, this work shows great potential for industrialisation.

## 1. Introduction

With the fast rise and development of human industry, waste gas, water, and solid pollution are becoming more severe, which poses a serious threat to human health [1,2]. Among them, heavy metal contamination has garnered significant attention from environmental experts and a huge amount of research has been conducted in recent years due to heavy metal bioaccumulation and non-degradability [3,4]. Chromium (Cr) is a typical heavy metal pollutant that is pervasive in the environment as a result of its broad usage in metallurgical, chemical, refractory, and cast-iron processes [5]. The predominant stable forms of chromium are trivalent chromium (Cr (III)) and hexavalent chromium (Cr (VI)). Cr (III) is a necessary element for the human body, and its toxicity is moderate [6]. The toxicity of Cr (VI), however, is about one hundred times that of Cr (III) [7], posing far greater environmental and health hazards. It can enter the human circulatory system through drinking water or food-chain enrichment and have harmful effects on people [8].

To solve the chromium pollution problem, many advanced treatment technologies such as reduction, ion exchange, adsorption, membrane filtration, and electrochemical treatment have been applied for efficient Cr (VI) removal [9]. Each method has its own benefits and drawbacks; for instance, adsorption can remove both Cr (III) and Cr (VI), but its capacity is limited [10]. Ion exchange is effective and simple to implement, but it is easily affected by other ions [11]. Membrane filtration can remove Cr (VI) on a large scale, but it is an expensive process [12]. Electrochemical treatment has a rapid reaction rate but produces an enormous amount of sludge [13]. Reduction requires only a one-step process, but it is unsuitable for drinking-water treatment [14]. Reduction and adsorption are the two most widely used methods among these. Integrating chemical reduction and adsorption together has proven to be more promising to increase Cr (VI) removal efficiency; consequently, several types of materials designed to achieve this have been widely investigated in recent years. For example, the non-toxic and cheap zero-valent iron (ZVI) has been proposed as a chemical precipitation agent to remove chromium contaminants [15,16,17,18]. Cr (VI) could be reduced by ZVI and form insoluble Cr (III) precipitates with a much lower toxicity in a neutral or alkaline environment. Previous studies have demonstrated that conventional micrometre-sized ZVI presents the ability to remove Cr (VI), but its relatively low adsorption capacity limits its application [19]. Nano zero-valent iron (NZVI) particles have a smaller particle size and a larger specific surface area than ordinary iron powders, showing a better Cr (VI) removal potential [20,21,22]. Although NZVI possesses great potential as a Cr removal agent, it does not work well in practical applications, mainly because of ageing [23], a lack of selectivity and directionality (e.g., reacts with nitrate and organic matter) [24], and agglomeration [25]. In addition, the separation of NZVI from the treated solution also presents a big challenge. All of these features limit the adsorption capacity and may produce secondary pollution [26]. In order to overcome the problem, researchers have tried to modify NZVI using a variety of different methods [27], such as forming polymetallic material [28] or support by an adsorbent [29].

Normally, polymetallic particles are constituted with a corrosive metal such as iron or zinc as a reductant along with a noble metal as a catalyst such as palladium (Pd) [30], copper (Cu) [31], nickel (Ni) [32], or cerium (Ce) [33]. Huang et al. [34] synthesised nickel–iron layered double hydroxide and modified the surface charge density to achieve a Cr (VI) removal capacity of 35.86 mg/g at an initial pH of 5. Li et al. [35] created a Cd_0.5_Zn_0.5_S/Bi_2_WO_6_ composite, which is capable of photocatalytically reducing Cr (VI) with a reduction efficiency of up to 95.8% at pH 5. Polymetallic materials have been proven to possess a higher Cr adsorption capacity and reaction rate compared to the pure NZVI system [36].

Certain limitations, however, also need to be solved including the aggregation problem that reduces its adsorption capacity [37], the secondary pollution of reduced Cr (III) as is not immobilized [38], and the difficulty of nanoparticle collection in practical application which may increase the operating costs [39]. Besides the polymetallic materials, porous-structured materials with a high specific surface area are proposed as excellent adsorbent candidates, such as clay minerals [40], bio sorbent materials [41], and carbon-based compounds [42]. Researchers have attempted to increase the Cr (VI) adsorption capacity by combining reducing metals or their oxides with adsorbents. For example, Zhou et al. [43] loaded nano zero-valent iron (nZVI) onto biochar made from recycled peanut shell waste; the composites achieved a 99.73 mg/g of Cr (VI) adsorption capacity at pH = 7.5. Bharath et al. [44] produced peanut-shell-derived activated carbon and its Fe_3_O_4_ nano-composite and demonstrated a maximum electrosorption capacity of 24.5 mg/g at 1.2 V.

It is believed that a better Cr removal effect should be achieved by integrating polymetallic and sorbent-supported material together. Previous studies have demonstrated that polymetallic loading increases the adsorption capacity by approximately 50% when compared to that of pure iron loading [45]. For example, Lu et al. [46] decorated an Fe–Ni bimetal on montmorillonite and obtained an adsorption capacity of about 65 mg/g at pH = 3. Zeng et al. [47] synthesized biochar-loaded Fe–Ni bimetallic particles and observed an adsorption capacity of 55.52 mg/g at pH = 3.1. Compared to natural materials, some carbon-based artificial materials such as reduced graphene oxide (RGO), graphene oxide (GO), and multi-walled carbon nanotubes (MWCNTs) are more promising because of their high surface area and purity. For example, Bharath et al. [48] synthesized a Ru–CoFe_2_O_4_/RGO photoelectrode with a maximum Cr (VI) reduction rate of 99.9% at 30 min when exposed to visible light. Kang et al. [49] loaded an Fe–Ni bimetal on RGO and achieved 197.43 mg/g at pH = 5. As two-dimensional RGO and GO could partly encapsulate metal particles and prevent them from reacting with Cr (VI), resulting in a decrease in Cr (VI) adsorption capacity and a low reusability, one-dimensional carbon materials should have a better performance compared with two-dimensional carbon materials [50]. Due to the unique tubular structure of MWCNTs and their extraordinarily high specific surface area, it is a suitable material for providing physical adsorption capacity as it ensures a high number of adsorption sites. Polymetallics such as nano iron and nickel, on the other hand, are an excellent choice for providing reduction capability. Integrating the above two materials together can ensure a target material with a high physical adsorption and chemical reduction capacity for Cr (VI); therefore, this is believed to achieve a promising Cr removal property. In the removal of Cr (VI), the following synergies are expected to occur: MWCNTs aggregate Cr (VI) in solution in the vicinity of the composite by physical adsorption; meanwhile, Fe rapidly reduces Cr (VI) to Cr (III) catalysed by Ni. MWCNTs also provide part of the Cr (VI) adsorption capacity based on their own physical adsorption. The chemical reduction capacity reduces Cr (VI) to Cr (III), which is less susceptible to re-oxidation in nature, preventing secondary contamination. The chemical reduction method will provide the majority of the Cr (VI) adsorption capacity.

Hence, this work aims to synthesize Fe–Ni/MWCNTs composite particles by loading Fe–Ni bimetallic particles onto MWCNTs through a co-precipitation and physisorption method. The adsorption capacity and reutilization properties were measured and analyzed in detail. The results indicate that the formed composite shows a higher adsorption capacity under similar conditions compared to those reported in the literature. A high adsorption capacity performance was observed for reuse. Considering the merits, including the low cost of all raw materials, simple synthesis process, and good reusability, the Fe–Ni/MWCNTs composites proposed here exhibit the potential for future industrialization.

## 2. Results and Discussion

### 2.1. Particle Size, Morphology, and Elemental Analyses

#### 2.1.1. Particle Size Analysis

**Figure 1:**
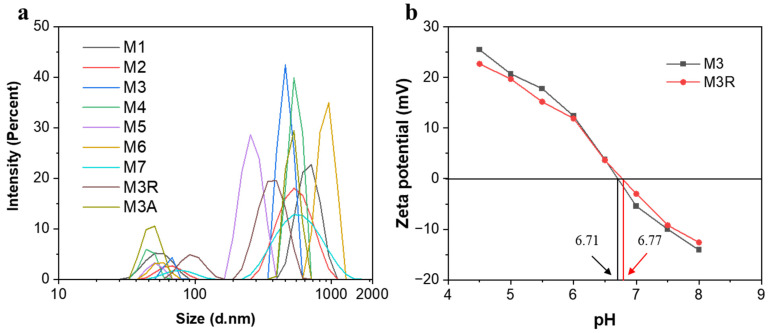
(**a**) Particle size distribution measured by DLS and (**b**) the zeta potentials of M3 and M3R at pH 4.5–8.0.

A centrifuge was used to separate the sediments from the liquid after adding 15 mg of the sample solution to 50 mL of deionized water under ultrasonic dispersion. The upper liquid was tested by Zetasizer, Malvern, and the results are shown in Figure 1a and Table A1. Two peaks were found in all samples. A larger size range indicates that MWCNTs have been stacked in an unordered manner. It is clearly shorter than the length of the MWCNTs (2500 nm) shown in the purchase company data, because the MWCNTs are curved rather than straight. The particles of about 50–80 nm in diameter should represent Ni and Fe (0) NPs. Figure 1b illustrates that the pHZPC values for M3 and M3R are 6.71 and 6.77, respectively, and their zeta potentials increase with decreasing pH values. At lower pH values, when the pH values are less than pHZPC points, the samples are better dispersed and stabilized, which has a positive effect on the adsorption capacity of Cr (VI). Furthermore, when the zeta potential is positive, the samples are positively charged, and the electrostatic effect enhances their attraction to the negatively charged Cr_2_O_7_^2−^ in solution.

#### 2.1.2. Morphology and Elemental Analyses

**Figure 2:**
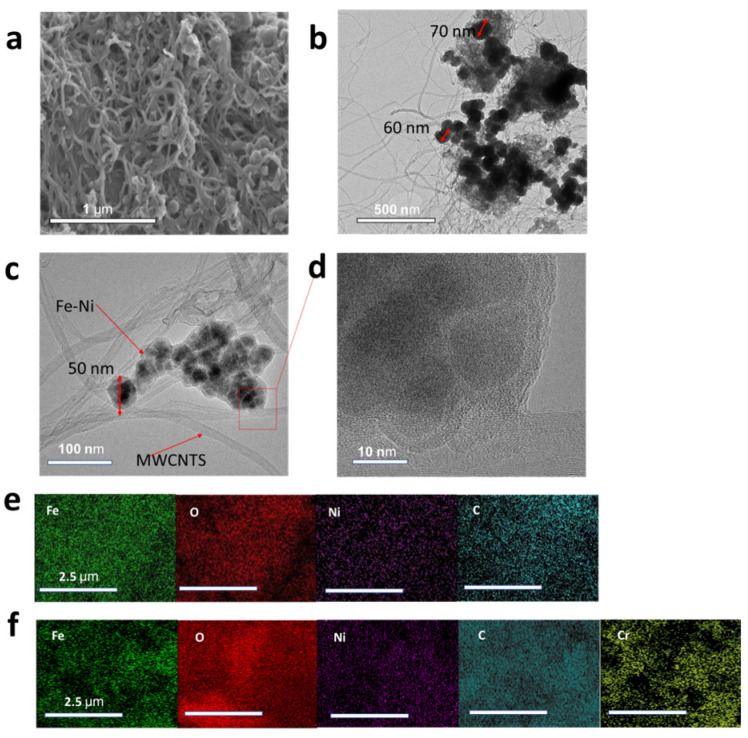
SEM image (**a**) and TEM image (**b**) for M3, TEM image (**c**) and HRTEM image (**d**) for M3A, and EDS image (**e**) for M3 and (**f**) for M3A.

TEM and SEM were used to examine the morphology and structure of the Fe–Ni/MWCNTs. The results in Figure 2 illustrate M3 as an unordered stacking of MWCNTs and Fe–Ni particles, and that the Fe–Ni bimetal particles are approximately 50–80 nm and the MWCNTs are more than 1 μm in diameter. This is consistent with DLS data. The energy-dispersive X-ray spectroscopy (EDS) results in Figure 2e illustrate the elemental composition of M3, indicating a uniform distribution of Fe, Ni, and C elements. Comparatively, Figure 2f illustrates that Cr appears after the sorption of Cr (VI), indicating that an amount of Cr element is immobilised on the surface of the sample. However, the valence state and compound structure need further characterization via other methods to confirm. The existence of O is due to metallic particles being inevitably partially oxidised, because nitrogen protection is not used throughout the whole synthesis process. For the M3A sample, the diameter of Fe–Ni particles decreased to around 50 nm after the Cr (VI) adsorption test. This is because part of the Fe (0) is consumed by Cr (VI) during the reduction process. Figure 2d illustrates how the Fe–Ni bimetal is decorated on the surface of MWCNTs in the HRTEM image for M3A.

**Figure 3:**
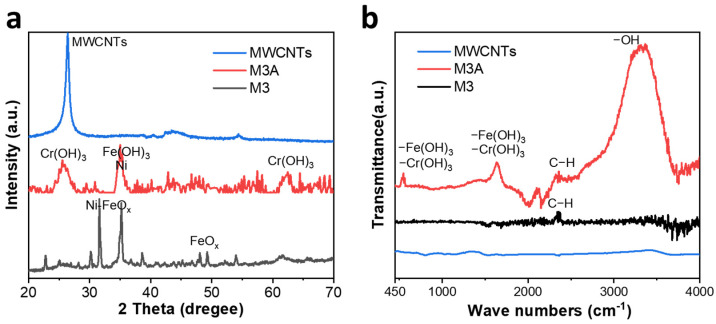
(**a**) XRD patterns and (**b**) FTIR spectra of unmodified MWCNTs, M3, and M3A.

Figure 3a illustrates the XRD patterns of unmodified MWCNTs, M3, and M3A. The unmodified MWCNTs exhibit a sharp peak at 26.3° corresponding to the typical (002) diffraction of MWCNTs (JCPDS No. 00–058-1638) [51]. Due to the covering of loaded materials, no similar sharp peaks were observed on M3 and M3A. The inevitable oxidation of Fe (0) [52] causes the double peaks at 31.6°, 35.2°, and 48.1°, 49.2° for M3 (JCPDS No. 01–072-6225). For M3A, the peak at 35.1° indicates the presence of Fe (OH)_3_ (JCPDS No. 00–022-0346) [53], and the peaks at 26.1° and 62.9° indicate the presence of Cr (OH)_3_ (JCPDS No. 00–012-0241) [54], both of which originate from the coordinated or adsorbed H_2_O in M3A. Figure 3b of the FTIR data demonstrates distinct peaks at approximately 550 cm^−1^ and 1623 cm^−1^, combined with the identified elements from the EDS results, that corresponds to Cr (OH)_3_ and Fe (OH)_3_ coprecipitated sediment [55,56]. The lack of an obvious peak observed around 900 cm^−1^ indicates that no considerable Cr=O exists [57,58]. This demonstrates that the sediment contains Cr (III) and Fe (III) in the form of Cr (OH)_3_ and Fe (OH)_3_, respectively. It is also consistent with the XRD results. Comparing M3A and unmodified MWCNTs, the distinct peak at around 3363 cm^−1^ represents the stretching and bending vibration of the -OH on MWCNTs in M3A [59]. The -OH is generated as a result of Cr (VI)’s strong oxidizing ability. The absence of a prominent peak for unmodified MWCNTs and M3 suggests that there are no considerable oxygen-containing groups on MWCNTs.

**Figure 4:**
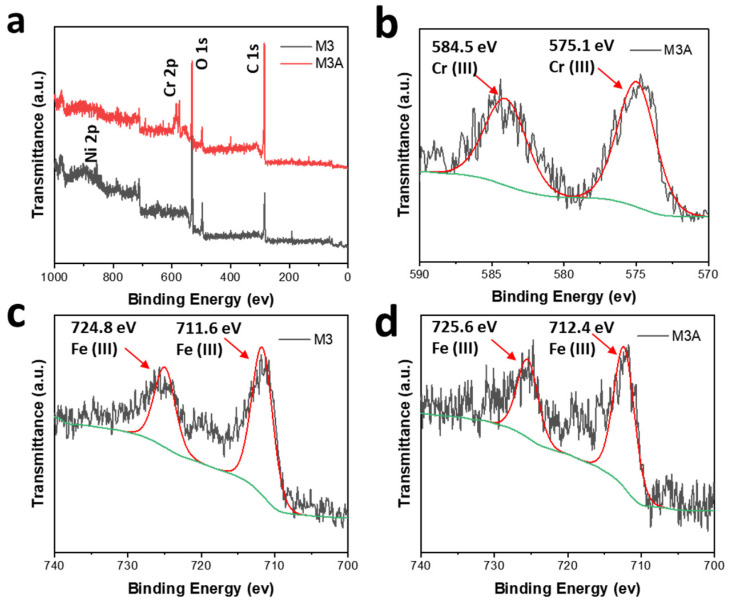
XPS spectra of M3 and M3A: (**a**) wide scan, (**b**) high resolution spectra of Cr in M3A, and high resolution spectra of Fe in (**c**) M3 and (**d**) M3A.

The XPS spectra in Figure 4a confirm that the M3 and M3A samples are primarily composed of C, O, Fe, and Ni. As shown in Figure 4b, the resolution spectra of the Cr 2p region magnifies the two deconvoluted peaks of Cr 2p1/2 and Cr 2p3/2. Based on the XRD and FTIR results above, the two peaks correspond to the Cr present in Cr (III) [60]. It confirmed that Cr (VI) is reduced to Cr (III) and adsorbed by the sample. In Figure 4c and Figure 4d, the peaks for Fe 2p are deconvoluted into two peaks. This is due to the presence of Fe_2_O_3_ in M3 and Fe (OH)_3_ in M3A [61]. The binding energy for Fe_2_O_3_ in M3 and Fe (OH)_3_ in M3A are nearly identical [62,63,64]. The results from XPS are consistent with those obtained from XRD and FTIR.

### 2.2. BET Surface Area Study

**Figure 5:**
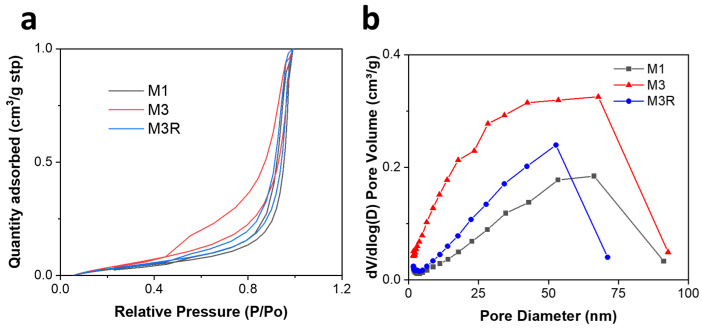
BET results of samples (**a**) N_2_ adsorption isotherms and (**b**) pore size distribution data.

The specific surface area of samples was measured by a Micromeritics Tristar 3000, as shown in Figure 5. According to the adsorption–desorption isotherm, the isotherm is a concave curve and has no inflexion point. The amount of gas adsorbed rises as the partial pressure of the components increases. The concave curve is due to the intermolecular interaction between adsorbates being stronger than that between adsorbent and adsorbates; the adsorption becomes self-accelerating as the process proceeds and exhibits multilayer adsorption characteristics [65,66]. M1, M3, and M3R have specific surface areas calculated as 23.6197 m^2^/g, 82.2434 m^2^/g, and 30.7797 m^2^/g, respectively. This indicates that the presence of nickel powder significantly increases the sample surface area, due to its own high surface area, and that it combines with Fe (0) to reduce the degree of Fe aggregation. In contrast, samples that contain nickel powder have a smaller distribution of pore widths. As a result of the aggregation of Ni (0) and Fe (0) during the Cr (VI) adsorption experiment and the second reduction process for Fe (III), the BET surface area of M3R was much smaller than that of M3. It should be noted, however, that the BET specific surface areas of all samples were considerably lower than those of pure MWCNTs (more than 200 m^2^/g). This is due to the disordered stacking of MWCNT, as indicated by the SEM results.

### 2.3. Cr (VI) Adsorption Capacity Analyses

**Figure 6:**
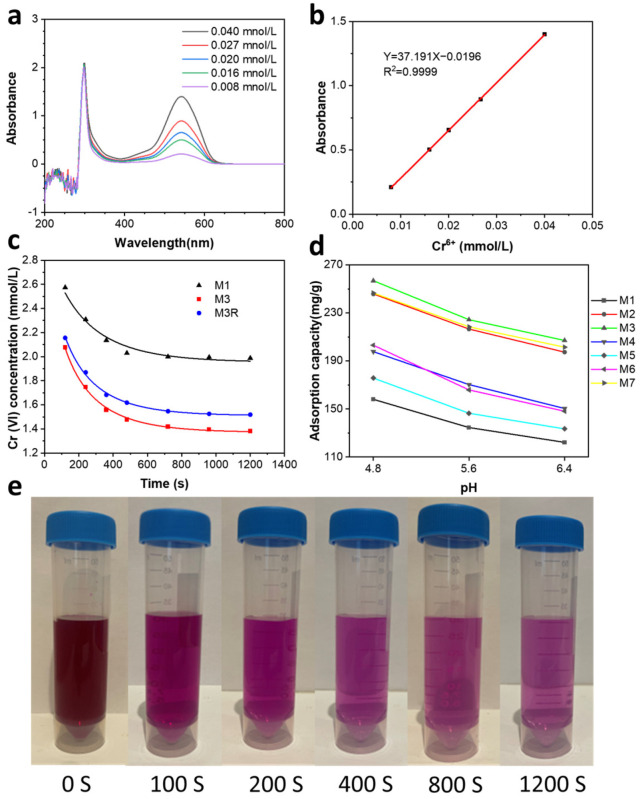
(**a**) UV–Vis spectra of solutions of Cr (VI) reaction with DPC; (**b**) calibration curve line of Cr (VI) concentration (λ = 545 nm); (**c**) Cr (VI) adsorption capacity at different pH values; (**c**) Cr (VI) adsorption capacity with time and pseudo-second-order kinetic model; (**d**) Cr (VI) adsorption capacity at different pH values; (**e**) violet color changes with time for Cr (VI) adsorbed by M3.

Figure 6a illustrates DPC combined with various Cr (VI) concentration solutions to produce a violet color solution with a characteristic peak at max = 545 nm. A linear relationship between Cr (VI) concentration and absorbance at λ_max_ = 545 nm is shown in Figure 6b. In the equation y = 37.191x − 0.0916 with R^2^ = 0.9999, x stands for the concentration of Cr (VI), while y stands for absorbance. Figure 6c illustrates that the Cr (VI) concentration stabilised after 20 min in most cases. Then, the adsorption capacity was calculated by analysing the remaining concentration of Cr (VI) (Table 1). Figure 6e shows the changes of strong violet color with time for Cr (VI) adsorbed by M3. The drastic diminishment of the violet indicates the decrease in Cr (VI) concentration.

Likewise, pure MWCNTs and NZVI were also tested under the same conditions for Cr (VI) adsorption capacity analyses. As shown in Table 1, pure MWCNTs and iron have very low Cr (VI) adsorption capacities. Fe (0)-decorated MWCNTs had a significantly higher adsorption capacity than pure MWCNTs and NZVI. The combined action of MWCNTs and NZVI was responsible for this result. Cr (VI) is adsorbed to the surface of MWCNTs by physical adsorption, and the adsorbed Cr (VI) is reduced by the decorated nano iron more effectively. Comparing M1 with other samples, it is clear that the addition of a certain amount of Ni can naturally increase the Cr (VI) adsorption capacity. Due to the high catalytic performance of Ni particles, the activation energy required for the reduction of Cr (VI) is decreased. From M2 to M7, different Cr (VI) adsorption capacities are obtained due to different Fe to Ni ratios. The ideal ratio for Fe–Ni is approximately 5:1, and M3 exhibits the highest adsorption capacity at 224.43 mg/g. 

**Figure 7:**
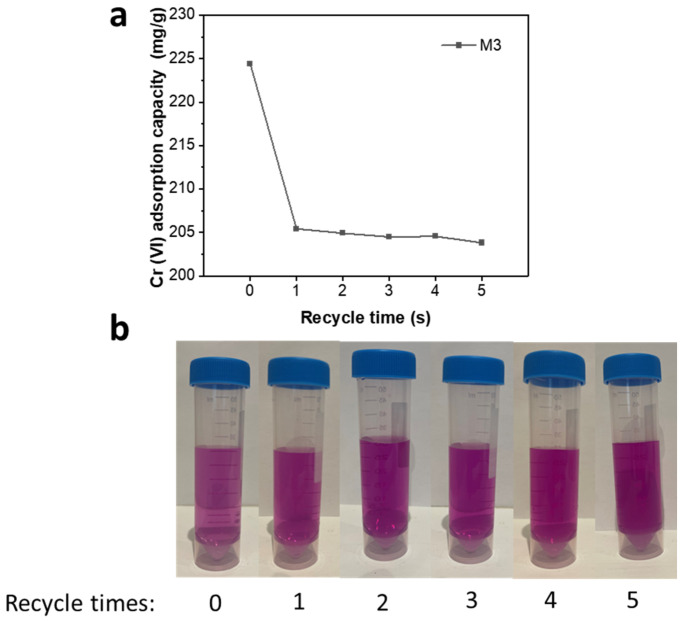
(**a**) Cr (VI) adsorption capacities for M3 with 1–5 replicates; (**b**) images of the violet color for Cr (VI) adsorbed by recycled M3.

The absorption capacities of M3 that has been recycled were examined 1–5 times. As shown in Figure 7a, in the Cr (VI) removal test for M3 with 1–5 replicates, the adsorption capacities are 205.41 mg/g, 204.91 mg/g, 204.48 mg/g, 204.55 mg/g, and 203.80 mg/g. Figure 7b shows the violet color of Cr (VI) adsorbed by recycled M3 for comparison. More than 90% of M3’s adsorption capacity is retained after five cycles of recycling, while there is no discernible decrease with increasing cycles. These data indicate that M3 is highly recyclable. In the initial synthesis of M3, iron ions were precipitation-adsorbed to the surface of the MWCNTs from liquid, resulting in nanoparticles with a smaller particle size and better dispersion. However, during the reprocessing of M3, the iron nanoparticles were converted from a trivalent solid precipitation, resulting in a larger average particle size and poorer dispersion. The different nanoparticle formation processes explain the small decrease in the adsorption capacity of M3 during the first reuse. Subsequent iterations showed little continued reduction in adsorption capacity, as the treatment process was identical.

The pH of the solution plays an important role in Cr (VI) adsorption. In order to determine how pH affects Cr (VI) adsorption, Cr (VI) adsorption experiments were conducted at pH = 4.8, 5.6, and 6.4. As shown in Table A2, the Cr (VI) adsorption capacity decreased as pH increased, which is consistent with previous research [67]. The highest adsorption capacity was observed for M3 at pH = 4.8 for 256.87 mg/g.

Furthermore, to verify the stability of samples, samples were left to stand in the solution for 3 months after the Cr (VI) adsorption experiment, and the Cr (VI) concentration in solution was characterized. The results are presented in Table A3. All samples remained stable and retained more than 93% of their adsorption capacity. This is because the Cr (III) hydroxide produced is stable and difficult to disperse back into the solution once it has been adsorbed by Fe–Ni/MWCNTs.

A comparison of Cr (VI) adsorption capacity with the reported MWCNTs or CNTs-related materials is shown in Table A4. As discussed previously, a higher pH has a negative impact on adsorption capacity; compared with the listed literature, this study demonstrates a significantly higher adsorption capacity for Cr (VI) even under negative pH effects. Under similar conditions, Fe–Ni/MWCNTs had a 20% higher Cr (VI) adsorption capacity than Fe–Ni/RGO. 

As shown in Figure 6c, the adsorption kinetics of Cr (VI) are obtained by determining the adsorption capacity of samples at different times. Initially, adsorption is rapid due to the enormous number of available sites, but it slows down with time and reaches equilibrium after 20 min. The results are analyzed using pseudo-first-order kinetic models and pseudo-second-order kinetic models. This pseudo-first-order kinetic model can be expressed linearly as follows:

$$\log\left(q_{e}-q_{t}\right)=logq_{e}-K_{1}t$$

The pseudo-second-order kinetic model can be expressed in linear form as follows:

$$\frac{t}{q_{t}}=\frac{1}{K_{2}q_{e}^{2}}+\frac{t}{q_{e}}$$

where $q_{e}$ and $q_{t}$ (mg/g) represent Cr (VI) adsorption capacity at equilibrium and t time (hour). $K_{1}$ (g/mg/min) and $K_{2}$ (g/mg/min) represent the pseudo-first-order kinetic model and pseudo-second-order kinetic model rate constants, respectively. The results of the analysis for the pseudo-first-order kinetic model and the pseudo-second-order kinetic model are displayed in Table 2. As compared with the pseudo-first-order kinetic model, the pseudo-second-order kinetic model obtained an adjusted R^2^ that is closer to 1, which indicates that the pseudo-second-order kinetic model is more appropriate for the samples. In this study, it is confirmed that the adsorption process is not physical but chemical in nature.

### 2.4. Cr (VI) Reduce Reaction Mechanism and Kinetic Model

**Figure 8:**
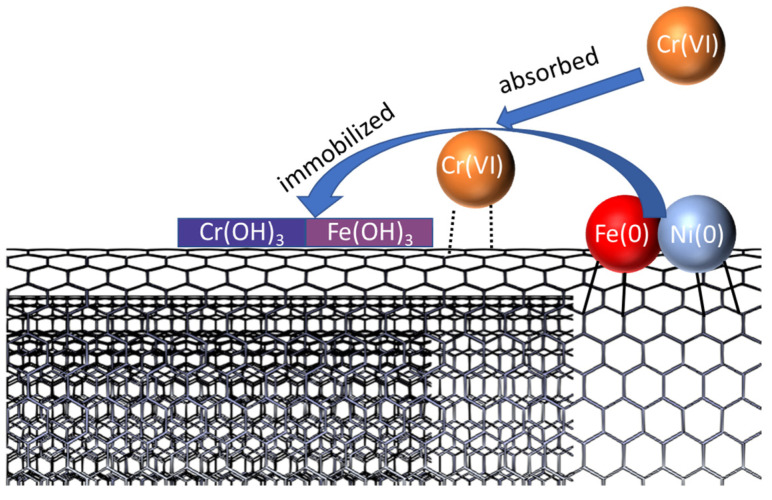
Cr (VI) adsorption and reduction mechanism of samples.

In this nano-composite, in combination with the dispersant, the spatial barrier effect of MWCNTs reduces the agglomeration of Fe–Ni bimetallic particles. It functions as an adsorbent to enrich the Cr (VI) surrounding the material, which increases the directionality of the chemical reduction capacity of the material and also provides part of the Cr (VI) adsorption capacity based on its own physical adsorption. Fe functions as a reducing agent to convert Cr (VI) to Cr (III), which is less susceptible to re-oxidation in nature, preventing secondary contamination. In the reduction process, Cr (VI) reacts with Fe (0) to form Cr (OH)_3_ and Fe (OH)_3_ precipitates. The added nickel has a higher reduction potential than Fe, which promotes the electron transfer of Fe (0) as the anode through electrochemical coupling, which enhances the reduction activity of iron nanoparticles; furthermore, the nickel nanopowder is also able to catalyse the hydrogenolysis reaction and improve the hydrogenation reaction of nano iron. These hydroxides precipitate and encapsulate Fe (0), preventing all iron from participating in the reaction, which is slowed down by the physical adsorption ability of the MWCNTs to adsorb some of the hydroxide precipitates onto its surface. The process is schematically illustrated in Figure 8.

All the formulae of the chemical reactions occurring in the Cr removal experiments are listed below:

$$2HCrO_{4}^{-}+14H^{+}+3Fe^{0}\left(s\right)\to 2Cr^{3+}+8H_{2}O+3Fe^{2+}$$

$$HCrO_{4}^{-}+7H^{+}+3Fe^{2+}\to Cr(III)+4H_{2}O+3Fe^{3+}$$

$$Fe^{0}+2Fe^{3+}\to 3{Fe}^{2+}$$

$$Fe^{0}+2H_{2}O\to {Fe}^{2+}+H_{2}+2OH^{-}$$

$$2Ni^{0}+H_{2}\to 2Ni^{0}-H\cdot$$

$$HCrO_{4}^{-}+3Ni^{0}-H\cdot +4H^{+}\to Cr(III)+{4H}_{2}O+3Ni^{0}$$

The major Cr (VI) status at a low pH is HCrO_4_^−^ and Cr_2_O_7_^2−^ [68]. The removal efficiency is greater at low pH values due to the highly protonated and positively charged surface of the adsorbent. Electrostatic force can attract HCrO_4_^−^ and Cr_2_O_7_^2−^ to the adsorbent. As the pH increases, the reaction rate and equilibrium are significantly affected by less $H^{+}$ and more $OH^{-}$. In addition, the surface of the adsorbent will be negatively charged, which drastically reduces its adsorption capacity.

**Figure 9:**
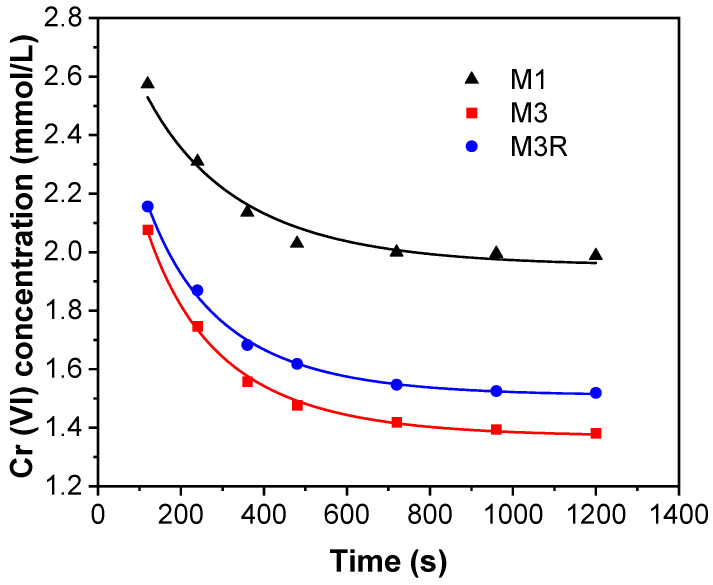
The kinetic model curve for M1, M3, and M3R.

As Cr (VI) adsorption is not a pure chemical or physical reaction, traditional pseudo-first-order kinetic models and pseudo-second-order kinetics cannot adequately describe it. All electrons are transferred from Fe species to Cr species; Fe is the only element capable of reducing Cr (VI). The rate equation for the Cr (VI) reduction is based on the concentration of Cr (VI) and the samples. The conversion ratio between Cr (VI) and Cr (III) is 1:1. A kinetic model can be applied [49]. The equation can be expressed as follows:

$$[Cr\left(VI\right){]}_{t}=\frac{\left[Cr\left(VI\right){]}_{0}\{C_{SC}^{*}\left[S\right]-[Cr\left(VI\right){]}_{0}\right\}}{C_{SC}^{*}\left[S\right]exp\{k(C_{SC}^{*}\left[S\right]-[Cr\left(VI\right){]}_{0}t)\}-[Cr\left(VI\right){]}_{0}}$$

where [Cr (VI)] represents the hexavalent chromium concentration (mmol/L) at time t; k represents rate coefficient (L mmol^−1^ min^−1^); $C_{SC}^{*}[S]$, represents the initial concentration of [SC]; [S] represents the sample concentration (g/L); $C_{SC}^{*}$ represents the removal compacity of Cr (VI) per unit gram of sample (mmol/g); k and $C_{SC}^{*}$ are the model constant parameters; and t represents the reaction time (s). The fit result of the kinetic model is shown in Figure 9 and Table 3. The kinetic model exhibits a high adjusted R^2^, which confirms that the reaction mechanism and model are reasonable for the reduction reaction. By comparing the rate coefficient *k* between M1 and M3, it is determined that Ni (0) added to M3 increased the reaction coefficient by a considerable amount, which is the result of Ni’s catalyst effect. In the meantime, comparing M3 and M3R, the decrease in K and $C_{SC}^{*}$ is due to the aggregation of Fe (0) and Ni (0) during the second reduction of Fe–Ni/MWCNTs.

## 3. Materials and Methods

### 3.1. Materials and Instruments

#### 3.1.1. Materials

MWCNTs and nickel (II) chloride hexahydrate were supplied by Sigma-Aldrich (Dorset, UK). FeCl_3_·6H_2_O and potassium dichromate (K_2_Cr_4_O_7_) were supplied by SLS (Scientific Laboratory Supplies). Polyvinyl pyrrolidone (PVP) and chromium (III) chloride hexahydrate (CrCl_3_) were purchased from Alfa Aesar. Sodium borohydride (NaBH_4_) was supplied by Fisher Scientific Ltd., and 1,5-diphenylcarbazide (DPC) was purchased from Sigma-Aldrich. The chemicals were used as received without further purification.

#### 3.1.2. Instruments

A scanning electron microscope (SEM, SU8230, Hitachi, Tokyo, Japan) equipped for energy-dispersive X-ray spectroscopy (EDS) was used to characterize the morphology, bonding patterns, and elemental distribution of the various components in a multi-component material, respectively. A transmission electron microscope (TEM, FEI Tecnai TF20) was used to observe the morphological changes in the components of the material before and after Cr (VI) removal tests to help analyse the reaction mechanism. Fourier-transform infrared spectroscopy (FTIR) experiments were performed with a Thermo Scientific Nicolet iS10 FTIR Spectrometer (Thermo Scientific, Waltham, MA, USA) to characterize the type and content of residual oxygen-containing groups on MWCNTs. BET surface areas were tested on a Tristar 3000 (Micromeritics) to analyze the effect of different components on the specific surface area and pore size distribution of the material. Ultraviolet–visible spectroscopy (UV–Vis) spectra of samples were measured by a UV spectrophotometer UV-1800 (Shimadzu, Kyoto, Japan) to quantify the concentration of Cr (VI) in each solution. Sample size characterization was investigated by dynamic light scattering (DLS) measurements using the Zetasizer Nano ZS (Malvern Panalytical, Malvern, UK). This was used to analyse the loading and agglomeration of metal particles. HAXPES UHV-XPS (X-ray photoelectron spectroscopy) was used to confirm the existence and the valence states of elements before and after the Cr (VI) removal experiment. 

### 3.2. Preparation of Fe/MWCNTs and Fe–Ni/MWCNTs Composites

The FeCl_3_ solution was formed by adding different amounts of 30 wt% FeCl_3_·6H_2_O solution to 50 mL of deionized water under magnetic stirring for 30 min, and then 0.32 g MWCNTs, 2.0 g PVP, and a certain amount of NiCl_2_·6H_2_O were added in sequence and magnetically stirred overnight. In different samples, Fe–Ni ratios were adjusted, but the total mass of Fe and Ni elements remained the same at 0.2 g. Afterwards, 40 mL of 50 mg/mL NaBH_4_ was added dropwise to the system and stirred overnight. As a reducing agent, NaBH_4_ reduced Fe (III) and Ni (II) to Fe (0) and Ni (0) NPs, respectively. During this period, mechanical oscillation replaced magnetic stirring in order to prevent Fe (0) NPs from being affected by the magnetic field. The production of H_2_ during this step requires careful control. Here are the reaction equations:

$${Fe(H_{2}O{)}_{6}}^{3+}+3B{H_{4}}^{-}+3H_{2}O={Fe}^{0}↓+3B(OH{)}_{3}+10.5H_{2}↑$$

$${Ni(H_{2}O{)}_{6}}^{3+}+3B{H_{4}}^{-}+3H_{2}O={Ni}^{0}↓+3B(OH{)}_{3}+10.5H_{2}↑$$

Following vacuum filtration, the final product was washed three times with ethanol, dried overnight in a vacuum oven, and then collected and stored with nitrogen protection. For the purpose of comparison, seven samples were prepared as shown in Table 4.

After the Cr (VI) adsorption capacity experiment of M3, the sediment was collected by vacuum filtration. The collected sediment was named M3A. Then, half of the M3A was dispersed in 25 mL DI water containing 1.0 g PVP under mechanical oscillation overnight. Afterwards, excess NaBH_4_ solution was added dropwise with mechanical oscillation. Then, the sediment was collected by vacuum filtration, washed with ethanol three times, and dried in vacuum oven overnight. The collected dried sediment was named M3R.

### 3.3. Cr (VI) Adsorption Capacity Test

The Cr (VI) adsorption capacity of samples was measured by the DPC method. In an acidic environment, DPC combines with Cr (VI) ions to produce a violet solution with a characteristic peak at max = 545 nm [69]. Prior to measurement, buffers with pH values 4.8, 5.6, and 6.2 were prepared using phosphoric acid (H_3_PO_4_) and sodium hydroxide solution (NaOH). An amount of 15 mg of each sample was added to 34 mL of pH buffer (pH = 5.6), followed by ultrasonic dispersion to ensure that the sample was fully dispersed. Afterwards, 6 mL of 20 mmol/L Cr (VI) solution was added to the solution to form a solution of Cr (VI) at a concentration of 3 mmol/L. The solution remained mechanically oscillated. At the scheduled time, 0.5 mL of solution was drawn and added to a bottle containing 2 mL of DPC solution and 22.5 mL of water. After 5 min, the UV absorption spectrum was measured to determine the amount of Cr (VI) remaining in the solution.

## 4. Conclusions

In this research, a novel Cr (VI) removal material was designed and produced comprising multi-walled carbon nanotubes (MWCNTs) as a support with a high specific surface area and Fe–Ni bimetallic particles as a catalyst loaded onto MWCNTs through the co-precipitation and physisorption method. Such a design produced composites with properties of high adsorption, reduction, and immobilisation of Cr (VI) for removal. Furthermore, they can be easily recollected and reused. The results indicate that the added Ni nearly tripled the BET surface area compared with samples without Ni, and added NI increased the Cr (VI) adsorption capacity significantly. Fe–Ni/MWCNTs composites exhibited the highest Cr (VI) adsorption capacity of 224.43 mg/g at pH = 5.6 and 256.87 mg/g at pH = 4.8, which are about twice those values reported for other materials under similar conditions. At least 93% of the adsorption capacity remained for reused samples, which demonstrates a good reusability. With a low cost of the raw materials, facile synthesis process, and good reusability, Fe–Ni/MWCNTs show great potential for future industrialisation.

## Figures and Tables

**Table 1:** The Cr (VI) concentration of samples deduced for 20 min.

	pH	Adsorption Percentage	Adsorption Capacity (mg/g)	Fe: Ni (Weight)
NZVI	5.6	5.42%	22.56	\
MWCNTs	5.6	4.80%	19.95	\
M1	5.6	32.35%	134.57	\
M2	5.6	52.07%	216.60	2:1
M3	5.6	53.95%	224.43	5:1
M4	5.6	40.95%	170.37	10:1
M5	5.6	35.22%	146.50	1:1
M6	5.6	39.88%	165.89	20:1
M7	5.6	52.52%	218.46	3:1
M3R	5.6	49.38%	205.41	5:1

**Table 2:** Pseudo-first-order kinetic model and pseudo-second-order kinetic model results.

	M1	M3	M3R
Pseudo-first-order kinetic model			
$q_{e}$ (mg/g)	149.9628	221.5793	203.0504
$K_{1}$ (g/mg/min)	0.1252	0.1755	0.1719
R^2^-adjusted	0.9860	0.9904	0.9855
Pseudo-second-order kinetic model			
$q_{e}$ (mg/g)	169.6151	249.8790	229.4719
$K_{2}$ (g/mg/min)	0.0020	0.0023	0.0024
R^2^-adjusted	0.9316	0.9766	0.9801

**Table 3:** The kinetic model results.

	M1	M3	M3R
K (L mmol^−1^ min^−1^)	0.0178	0.1734	0.1650
$C_{SC}^{*}$ (mmol/g)	2.8162	4.3445	3.9733
R2-adjusted	0.9981	0.9974	0.9972

**Table 4:** Detailed reaction conditions of different samples.

Sample	60 wt% FeCl_3_ Solution (mL)	MWCNTs (g)	NiCl_2_·6H_2_O (g)	PVP (g)	NaBH_4_ (g)	Fe: Ni (Weight)
M1	1.033	0.320	\	2	2	\
M2	1.278	0.320	0.534	2	2	2:1
M3	1.606	0.320	0.268	2	2	5:1
M4	1.760	0.320	0.156	2	2	10:1
M5	0.968	0.320	0.808	2	2	1:1
M6	1.839	0.320	0.078	2	2	20:1
M7	1.452	0.320	0.404	2	2	3:1

## Data Availability

Not applicable.

## Sample Availability

Samples of the compounds Fe–Ni/MWCNTs are available from the authors.

## Appendix A

Peak 2 has a smaller diameter than M3 for M3A. This is due to Fe (0) being consumed by Cr (VI) during the reduction process of Cr (VI). In addition, Peak 2 intensity for M3A increased from 6.9% for M3 to 32.4%. This is indicative of Fe–Ni bimetal falling off the surface of MWCNTs following Cr (VI) reduction. As can be seen from Table A1, Peak 2 for M3R is larger than Peak 2 for M3 and M3A. As a result, Fe–Ni nanoparticles agglomerate during the second reduction of Fe–Ni/MWCNTs. It is anticipated that the agglomeration of Fe–Ni nano iron will negatively affect the adsorption capacity of Cr (VI). The intensity of Peak 2 for M3R decreased from 32.4% to 16.9%, indicating that part of the Fe–Ni bimetal that was lost in M3A was decorated back on the surface of MWCNTs during the second reduction of Fe–Ni/MWCNTs. The reusability of samples can be enhanced by this method.

**Table A1:** Size distribution determined by the intensity.

	Peak 1		Peak 2	
	Size (d. nm)	% Intensity	Size (d. nm)	% Intensity
M1	684.3	79.1	54.85	20.9
M2	555.0	90.7	66.46	9.3
M3	461.5	93.1	64.91	6.9
M4	544.2	86.6	46.01	13.4
M5	261.5	92.6	51.31	7.4
M6	919.7	91.2	55.24	8.8
M7	605.1	92.0	78.95	8.0
M3R	370.0	83.1	96.96	16.9
M3A	519.5	67.6	48.06	32.4

**Table A2:** pH effects on Cr (VI) adsorption.

	Adsorption Percentage			Adsorption Capacity (mg/g)		
	pH = 5.6	pH = 4.8	pH = 6.4	pH = 5.6	pH = 4.8	pH = 6.4
M1	32.35%	38.00%	29.38%	134.57	158.06	122.24
M2	52.07%	40.91%	30.74%	216.60	245.87	197.40
M3	53.95%	59.10%	47.45%	224.43	256.87	207.09
M4	40.95%	61.75%	49.78%	170.37	197.77	150.42
M5	35.22%	47.54%	36.16%	146.50	175.77	133.45
M6	39.88%	42.25%	32.08%	165.89	203.18	148.18
M7	52.52%	48.84%	35.62%	218.46	246.61	201.50

**Table A3:** Sample stability for Cr (VI) adsorption.

	pH	Adsorption Percentage	Adsorption Capacity (mg/g)	After 3 Months	Adsorption Capacity Left
M1	5.6	32.35%	134.57	131.03	97.37%
M2	5.6	52.07%	216.60	205.97	95.09%
M3	5.6	53.95%	224.43	217.16	96.76%
M4	5.6	40.95%	170.37	164.59	96.61%
M5	5.6	35.22%	146.50	141.84	96.82%
M6	5.6	39.88%	165.89	155.83	93.93%
M7	5.6	52.52%	218.46	212.68	97.35%

**Table A4:** Cr (VI) adsorption capacity reported in the literature.

Raw Material	pH	Adsorption Capacity (mg/g)	References
CNTs supported by activated carbon	2.0	9.0	[70]
ionic liquid functionalized oxidized MWCNTs	2.8	85.83	[42]
MWCNTs-COOH-immobilized HSO_4_	2.0	31.29	[71]
Magnetic iron oxide MWCNTs	3.0	12.61	[72]
FeMnO_x_ decorated MWCNTs	2.0	47.25	[73]
α-Fe_2_O_3_/MWCNTs	6.0	Around 75	[74]
ZnO-functionalized MWCNTs	2.0	Up to 140	[75]
Fe–Ni/RGO	6.0	176.74	[49]
Fe–Ni/RGO	5.0	197.43	[49]
Fe–Ni/MWCNTs	4.8	256.87	This work
Fe–Ni/MWCNTs	5.6	224.43	This work
Fe–Ni/MWCNTs	6.4	207.09	This work
